# Magnetic Resonance Techniques Applied to the Diagnosis and Treatment of Parkinson’s Disease

**DOI:** 10.3389/fneur.2015.00146

**Published:** 2015-07-03

**Authors:** Benito de Celis Alonso, Silvia S. Hidalgo-Tobón, Manuel Menéndez-González, José Salas-Pacheco, Oscar Arias-Carrión

**Affiliations:** ^1^Facultad de Ciencias Físico Matemáticas, Benemérita Universidad Autónoma de Puebla, Puebla, Mexico; ^2^Fundación para el Desarrollo Carlos Sigüenza, Puebla, Mexico; ^3^Departamento de Imagenología, Hospital Infantil de México “Federico Gómez”, Mexico City, Mexico; ^4^Departamento de Física, Universidad Autónoma Metropolitana Iztapalapa, Mexico City, Mexico; ^5^Unidad de Neurología, Hospital Álvarez-Buylla, Mieres, Spain; ^6^Instituto de Investigación Científica, Universidad Juárez del Estado de Durango, Durango, Mexico; ^7^Unidad de Trastornos del Movimiento y Sueño (TMS), Hospital General Dr. Manuel Gea González, Mexico City, Mexico

**Keywords:** Parkinson’s disease, neuroimaging, MRI, TMS, diffusion MRI

## Abstract

Parkinson’s disease (PD) affects at least 10 million people worldwide. It is a neurodegenerative disease, which is currently diagnosed by neurological examination. No neuroimaging investigation or blood biomarker is available to aid diagnosis and prognosis. Most effort toward diagnosis using magnetic resonance (MR) has been focused on the use of structural/anatomical neuroimaging and diffusion tensor imaging (DTI). However, deep brain stimulation, a current strategy for treating PD, is guided by MR imaging (MRI). For clinical prognosis, diagnosis, and follow-up investigations, blood oxygen level-dependent MRI, DTI, spectroscopy, and transcranial magnetic stimulation have been used. These techniques represent the state of the art in the last 5 years. Here, we focus on MR techniques for the diagnosis and treatment of Parkinson’s disease.

## Introduction

Parkinson’s disease (PD), a movement disorder related to dopamine insufficiency in the brain, affects at least 10 million people worldwide. PD is one of the most complex neurodegenerative diseases, with a broad spectrum of motor and non-motor symptoms (1). According to the United Kingdom Parkinson’s Disease Society Brain Bank, clinical diagnosis is based on the presence of two or three motor features: bradykinesia, plus rigidity, or tremor at rest (or both). However, these criteria do not separate PD from the many other forms of parkinsonism, nor do they consider the non-motor symptoms. Despite extensive research, no effective therapy is currently available to prevent the onset of the disease, or to halt its progression (2).

**Figure 1:**
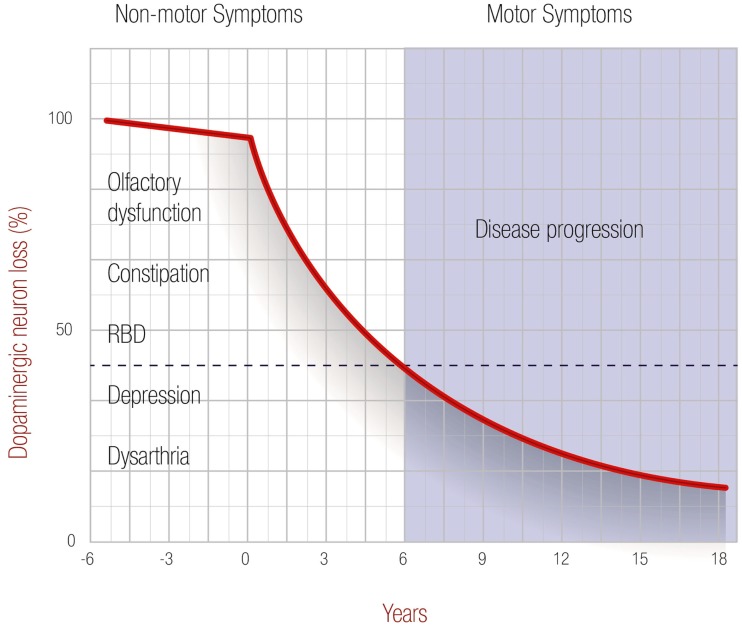
**Progression of Parkinson’s disease**. Degeneration of dopaminergic neuronal loss is correlated with motor and non-motor symptoms in PD patients. The graph starts at a time 0 when dopaminergic neuronal loss starts, and ends after 18 years, which was selected arbitrarily as no large physiological changes are expected after 10 years. When motor symptoms increase, significant neuroimaging differences can be found using MR and TMS techniques.

There is growing evidence that non-motor symptoms may precede motor symptoms – and a Parkinson’s diagnosis – by years. As the disease progresses and dopaminergic neurons continue to be lost, the motor symptoms appear and neuroimaging is useful for diagnosis, but by then it is too late for neuroprotection. No specific life expectancy prediction exists for PD patients after confirmation of the disease, but mortality rates are double those of healthy individuals at the same age. The relationship between dopaminergic neuronal loss and non-motor and motor symptoms is shown in Figure 1. This image is based in an extensive review in the field as well as some of the images and results from Shapira et al. (3). We use a time span for progression of 18 years, which is an arbitrary choice, as survival will be unpredictable at these stages of the disease.

Magnetic resonance (MR) technologies are based on the use of magnetic fields and radio-frequency pulses to obtain anatomical images. Imaging is the most common application of MR, but the strength of this technology lies in the fact that many other measurements can be made. Some examples of these methods are presented in this review, including blood oxygen level-dependent functional magnetic resonance imaging (BOLD-fMRI, a measurement of hemodynamics), perfusion (the process involved in the delivery of nutritive blood to the brain tissue capillary bed), and spectroscopy (measurement of the chemical content of a tissue). We also discuss transcranial magnetic stimulation (TMS) technologies, including simple pulse and repetitive TMS (4–6).

To date, published data support the use of MR imaging (MRI) for the diagnosis and treatment of PD (7, 8). There is also a large amount of work published on MR techniques, such as resting states fMRI (RS) and diffusion tensor imaging (DTI), as well as functional connectivity changes in the presence of the disease [e.g., Ref. (9, 10)]. Several systematic reviews have addressed the treatment applications for PD that may be combined with MR techniques. There are two primary ones, namely, focused ultrasound (11) and deep brain stimulation (DBS), both guided by MRI (12). To complement all of this excellent previous work, we address those additional techniques, which are not mainstream applications of MR to PD. Data from these studies inform neuroimaging, clinical follow-up, and possible intervention to treat PD. Here, we review highly specialized MR-based techniques for the diagnosis and treatment of PD.

## Methods

We followed the PRISMA model (Preferred Reporting Items for Systematic reviews and Meta-Analyses, http://www.prisma-statement.org/). We performed a search of articles cited in PubMed, Web of Science, Scopus, and SciELO from 2009 to 2014 using the following MeSH terms (Medical Subject Headings): “Parkinson’s disease” and “drug therapy,” “blood oxygen level dependence,” “functional magnetic resonance imaging,” “diffusion tensor imaging,” “spectroscopy,” or “transcranial magnetic stimulation.” We found a total of 424 studies related to PD and these MR/TMS techniques. After filtering publications using all the criteria previously introduced, 61 articles remained for these terms, which are presented and discussed in this study. In addition, we included nine articles published before 2009, since they pioneered the topic and are necessary for our discussion.

## Results

### BOLD-fMRI studies in parkinson’s disease

Blood oxygen level-dependent functional magnetic resonance imaging is a technique, which, in general terms, gives information about regions that are activated in the brain when performing a task. This information is obtained by measuring the hemodynamic changes produced in the activated region due to neuronal activity. The main limitation of this technique is that the relationship between neural activity and blood oxygenation has not been completely characterized. It is also significantly limited by the temporal resolution of the measurements, as they depend on hemodynamic effects (changing on the order of seconds), which are slow compared to neuronal activations (order of milliseconds). As one might suspect, due to the etiology of PD, the areas most hypothesized to be affected are those related to the dopaminergic system, specially the substantia nigra, as well as all motor regions (13).

Blood oxygen level-dependent functional magnetic resonance imaging studies for PD over the last 5 years can be divided into two groups. First, there are studies that assess the effect of medication on BOLD activation. Second, there is research focused on task-related experiments, which highlight the brain areas affected by PD.

### Effect of medication on BOLD-fMRI activation in PD

It is important to know, when a task-based BOLD-fMRI experiment is performed, the confounding effects that patients under medication may be subject to. Even though BOLD mechanisms are not perfectly described, they are known to be affected by changes in the flow and volume of fresh and deoxygenated blood. Common drugs can be used to investigate brain activation in PD patients. Levodopa corrects dopamine depletion in the brains of PD patients, by increasing dopamine concentrations. In contrast, apomorphine is a dopamine agonist, which binds to the corresponding receptors, creating a detectable electrical signal for this neurotransmitter. There is a large body of results, which shows that levodopa does, in fact, affect hemodynamic parameters. An example of this research is the paper by Kraft et al., which shows how the thalamus and the putamen have reduced BOLD responses in the absence of levodopa, but return to baseline with it (14). However, this effect was not found in cortical structures. Furthermore, Buhmann et al. showed that BOLD activation in the primary and supplementary motor areas (SMAs) increases in patients under levodopa, when compared to drug-naive patients (15). This increase was ratified by Ng et al., with the extra finding of changes in the spatial extent of BOLD activation, which were similar between controls and levodopa PD patients (16). Finally, in recent work by Martinu et al., medication with levodopa was found to increase corticostriatal motor network activations, during finger tap movement, in a medicated group compared to a medication-free group (17). Apomorphine studies showed an inverted effect, relative to levodopa, with reduced activation in the contralateral precentral gyrus when performing a tapping task after drug administration (18). Even though the majority of research points toward a large effect of medication on BOLD findings in PD, there is some evidence against it. The limitation of these investigations lies in the fact that they are focused on a hypercapnic model. Similar vasoreactivity to hypercapnia for patients with and without levodopa treatment has been observed (19).

### BOLD-fMRI task experiments in parkinson’s disease: Areas activated

The most common tasks used in PD studies with BOLD-fMRI are presented in Table 1. Almost all pharmacological studies presented in the previous section, and those found in the literature, used the finger tapping task, which makes it the most-used protocol to study BOLD-fMRI activation in PD (20–29).

**Table 1:** **Task-based BOLD-fMRI studies focusing on the brain areas affected in Parkinson’s disease**.

Task	Group description	Activity of group 1 higher that group 2	Activity of group 1 low that group 2	Reference
Tactile stimulation	PD patients (1) vs. healthy control volunteers	R. primary sensory, R. motor cortex, R. supplementary motor area, B. caudate, B. precuneus, B. occipital visual cortex, L. and M. temporal gyrus		(23)
Time perception task	PD patients (1) vs. healthy control volunteers and medical patients (2)	Precuneus (after long stimuli)		(26)
Gambling	Non-Pathological gamblers whit PD (1) vs. Pathological gamblers with PD (2)	B. anterior cingulate cortex, M. and S. frontal gyri, precuneus, R. I. parietal lobule, ventral striatum		(24)
Imaginary gait	PD patients experiencing freezing (1) vs. PDs not experiencing freezing (2)	*R. globus* pallidus, R. supplementary motor area, R. mesencephalic locomotor region, R. cerebellum, B. anterior insula, B. ventral striatum, B. pre-supplementary motor area, L. sub-thalamic nucleaus		(28, 29)
Hand movement	PD patients with (1) and without (2) mirror movements syndrome	R. dorsolateral prefrontal cortex, M. prefrontal cortex, Pre-supplementary motor area, Occipital Cortex	*B. insula*, B. posterior cingulate cortex, L inferior frontal cortex	(27)
Balloon analog risk task	PD patients with an impulse-control disorder (1) vs. PD patients without and impulse-control disorder (2)	R. ventral striatum		(25)
Speech	PD patients (1) vs. healthy control volunteers	Primary orofacial sensorimotor cortex		(20)
Saccade	PD patients (1) vs. healthy control volunteers	Frontal and supplementary eye fields	L. and R. cerebellum contralateral motor cortex	(22)
Finger tapping	PD patients (1) vs. healthy control volunteers	Putamen, supplementary motor area	R. prefrontal cortex, R. caudate	(21)

*Here, we show the results from BOLD-fMRI studies performed on PD patients in the last few years. Data are presented in five columns. The task and ability involved appears first. A description of the groups compared is presented in column two. Column three shows areas of the brain with significant differences, which were smaller for the first group vs. the second. The fourth column is similar to the third but with an inverted comparison. The last column presents the author and year of publication*.

*L, left; R, right; B, bilaterally; M, medial; S, superior*.

The SMA is known to be involved in modulating bimanual movements and sequencing motion, while the pre-supplementary motor area (pSMA) is known to connect the prefrontal cortex with the spinal cord. If etiological and pharmacological information is merged with the (real or imaginary) motion studies outlined in Table 1 for PD patients, both structures show an activation deficit. These results were logical, and accounted for the movement disorder component of PD. A new motion task (more complicated than finger tapping), which was being used to detect PD in its early stages, also showed hypoactivation in motor regions, such as the cerebellum, and bilaterally in the SMA (30). The relevance of this last study was that the reduced cortical activity in primary motor cortex and SMA in PD patients did not initially relate to motor symptoms (31).

The precuneus is known to be involved in memory, self-awareness, consciousness, and visuospatial function (attention detection). The precuneus was a region found to have weaker activations in PD patients during a series of different tasks (tactile stimulation, decision-making, and time perception) (13, 32). This region was not expected to be activated, as it did not belong to the group of basal ganglia and motion structures affected by PD. Hypo-activations in the precuneus were observed during tasks, which did not require motion (33). These studies detected a deficit in retrieval of episodic and contextual memories, which are useful for interpretation of tactile stimuli as well as decision-making and awareness of the passage of time.

The main message we can draw from this section is that, as expected, almost all research demonstrated at least one substructure of the basal ganglia to be hypoactive during BOLD-fMRI studies. This hypoactivity was also shown in brain structures related to motion. When other structures produced a hypoactive BOLD signal, it was mainly due to the stimulation protocol used. This was the case for the precuneus and the ventral striatum during texture or gambling protocols. Finally, all of these findings must be taken cautiously, as medication was found to influence the enhancement or reduction of BOLD activity.

### Perfusion studies in parkinson’s disease

Magnetic resonance perfusion measurements are techniques, which use contrast agents or the magnetic properties of blood to measure flow, transit time, time to peak, and volume of blood to a given tissue over time. There are three techniques used in MR: two with gadolinium as a contrast agent – dynamic susceptibility and dynamic contrast enhanced MR perfusion – and another with arterial spin labeling (ASL), which uses blood as the contrast agent (34). For details of some aspects of this technology see Ref. (35). New methods, more specific than traditional ASL, are now starting to become available. Even though perfusion techniques for clinical diagnosis and treatment monitoring are still in their infancy, the precision of new MR perfusion methods now rivals that of nuclear medicine or computed tomography. In the near future, these new techniques are expected to complement and overtake nuclear medicine, as they do not need nuclear radiotracers or artificial contrasts (36), making clinical studies non-invasive, and easy to perform.

Differences in blood perfusion have already been reported in multiple brain regions. For example, hypoperfusion was observed in the posterior cortex for both non-demented and demented PD patients, when compared to controls (37). Rao et al. also showed that flow to the right ventral striatum (a reward hub) diminished in PD patients with an impulse-control disorder, when compared to those without it (25). In a recent study by Fernández-Seara et al., in which absolute cerebral blood flow was measured with ASL techniques, hypoperfusion was found all over the cortex, and in some subcortical areas, such as the caudate nucleus (38). As the whole brain was investigated, and no global mean normalization was performed on the data, the possibility of artifacts due to this correction was negligible.

Pulsed arterial spin labeling (PASL), pseudocontinuous arterial spin labeling (PCASL), and continuous arterial spin labeling (CASL) are techniques, which apply a continuous RF pulse during which there is a continuous inversion of the arterial blood. An example of this was the work by Ma et al., in which CASL was used to measure the disease-related spatial covariance pattern of PD patients (25). This parameter was elevated in PD patients. When compared to standard ^18^F-fluorodeoxyglucose positron emission tomography studies, similar results were found. Another study using CASL techniques has also showed promising results in the early diagnosis of parkinsonian patients (39). The authors showed decreased cortical perfusion bilaterally to the posterior and inferior parietal, temporal, insular, lateral occipital, and prefrontal association cortices. Some increases were also seen in the cerebellum, pons, pallidum, right thalamus, sensorimotor cortex, SMA, and paracentral lobule.

It has also been shown that the differences in brain blood flow between patients with Alzheimer’s disease (AD) and those with PD and dementia are not that major, suggesting a closely linked mechanism for neurodegeneration (translational neurodegeneration). Absolute blood flows were similar for both groups, although hypoperfusion was found in the posterior cingulate gyrus, precuneus, and occipital regions, when the disease groups were compared with controls. PD patients had greater perfusion in the temporal lobes compared to AD patients, while the latter had greater perfusion in the right frontal cortex (40).

Finally, in work from Brusa et al., which performed perfusion-weighted dynamic susceptibility imaging, MR studies of PD patients showed that the effect of apomorphine medication on perfusion was to return the parameters toward the values obtained from control volunteers (41).

### Pharmacological techniques meet MR technology

Other applications of perfusion methods are pharmacological tests, which assess the uptake of a drug and its effects in patients. Perfusion MRI can provide rapid, quantitative, clinically relevant dose-finding information for pharmaceutical development. In one example of a pharmacological study in the PD field, an adenosine A2a antagonist (SYN115) was given to patients under levodopa but with no other antagonists. SYN115 produced a decrease in thalamic cerebral blood flow. Similar decreases occurred in cortical regions whose activity decreased with increased alertness and externally focused attention (42).

In conclusion, regions matching those found using BOLD-fMRI were observed to be hyper- or hypoperfused in perfusion studies. In general, the areas, which presented lower BOLD signals in PD patients, were found to be less perfused than in controls. This correlation is to be expected, as cerebral blood flow and cerebral blood volume are vital components of the BOLD signal. As before, medication had a confounding effect on the results obtained with this technique. An advantage of MR perfusion over BOLD-fMRI was that results were more centralized in the parenchyma of the brain, rather than its vascularity. As results continue to pile up, it is becoming obvious that findings from nuclear medicine are being closely matched and mirrored by MR perfusion techniques.

### Spectroscopy studies in parkinson’s disease

Nuclear magnetic resonance spectroscopy (MRS) is performed in spectrometers, which differ from normal MR scanners in that they lack magnetic gradients. MRS techniques do not produce images, but give information about the presence and quantity of a particular chemical in a given piece of tissue. For this reason, they are mainly used to monitor metabolic changes and serve as non-invasive biopsies. Some of the most common species measured are ^1^H, ^31^P, ^13^C, and ^23^Na. MRS can also measure more complex compounds, such as creatine, glucose, alanines, and lactates (present in some kinds of tumors). For the specific case of PD, the concentration of *N*-acetyl aspartate (NAA) is a powerful biomarker for assessing neuronal loss (12), while choline gives a good idea of membrane turnover, and creatine (Cr) can be used as a proxy for energy metabolism. Other parameters, like the ratio of choline (Cho) to Cr, the NAA-to-Cr ratio, GABA and phosphorus (associated with molecules of adenosine triphosphate) are also used for spectroscopy studies of PD. The down-side to these techniques is the need for very strong magnetic fields, in order to obtain measurable signals from compounds, which are much less abundant than the ^1^H species measured in normal MRI. This limits the hardware and technology available as well as the availability, and hence usefulness of the technique.

There is a large body of work using this technique in PD. We present some of the most recent findings in Table 2. The table shows that results obtained from MRS should be further divided, considering possible comorbidities as well as the stage at which the PD patients were studied. For example, NAA-to-Cr ratios have been found to be reduced in the SMA, when compared to healthy volunteers (43). All these results were furthered, showing that these differences were not significant when comparing controls with patients at an early stage of PD (44). Nevertheless, motor function improved after 6 months of treatment with pergolide (dopamine agonist). This improvement was correlated with the increase of the Cho/Cr ratio found in motor areas (45). Another example is the comparison of PD patients with or without dementia. In this case, the concentration of NAA in the occipital lobes was reduced in the demented group, when compared to non-demented PD patients Fayed (46).

**Table 2:** **Spectroscopic studies in Parkinson’s disease**.

Group description	Affected region	Chemical or ratio studied	Variation between group 1 and 2	Reference
Control (Group 1), PD patients (2)	SMA	NAA:Cr	Reduced in PD patients	(43)
Control (Group 1), PD patients (2)	Motor cortex	Cho:Cr and NAA:Cr	Reduced Cho:Cr and NAA:Cr in PD patients. Differences in the Cho:Cr tend to disappear after dopamine treatment	(45)
Control (Group 1), PD patients (2)	Posterior cingulate gyrus	Glu:Cr, NAA:Cr, Cho:Cr	Reduced Glu:Cr in PD patients	(47)
PD patients with mild cognitive impairment (Group 1) Normal PD patients (Group 2)	Posterior cingulate gyrus, occipital lobe	NAA:Cr	Reduced NAA:Cr for PD patients with mild cognitive impairment	(48)
Control (Group 1), PD patients (2)	Anterior and posterior cingulate cortex	NAA:Cr	Reduced in anterior cingulate cortex for PD patients. Not in posterior cingulate cortex	(49)
Control (Group 1), PD patients (2)	Substantia nigra	NAA:Cr	Reduced NAA:Cr for PD patients	(50)
Control (Group 1), PD patients (2)	Substantia nigra	NAA:Cr, NAA:Cho, NAA:(Cho + Cr)	All ratios reduced for PD patients vs. control. All ratios reduced damage hemispheres of PD patients vs. healthy hemisphere	(51)
Control (Group 1), PD patients (2)	Putamen, pons	GABA	Higher for PD patients vs. control	(52)

*Here, we show results based on spectroscopy in PD patients in the last few years. Data are presented in five columns. The groups compared appear first. The brain area studied is presented in column two. Column three shows the metabolite or the ratio studied. The fourth column shows the differences in this ratio or metabolite between groups. The last column presents the author and year of publication*.

In a recent study, spectroscopy techniques were applied not to a given region but to the whole brain. Here, NAA, total Cr, and total Cho were measured and ratios were calculated for the white and gray matter, as well as for each brain lobe. The main finding was that a general increase in Cr for PD patients was observed in an early stage of the disease, when compared to controls. The authors hypothesized that this result reflected a compensatory greater neuronal energy expenditure in early PD Levin (53).

A large body of work has been focused on the cingulate cortex (47–49) as well basal ganglia structures (50–52). Nevertheless, few studies have focused on other regions also known to be affected in PD. Some of these regions would be the precuneus, cerebellum, insula, and the pre- and postcentral gyri. Current trends in MRS research are toward combining the findings in this field with results from other MR techniques. An example of this is the research from Modrego et al., where the authors compared a PD rating scale with DTI and glutamate and glutamine concentrations obtained from MRS in the lenticular nucleus (54). They found that the combined increase of glutamate and glutamine in the lenticular nucleus in PD patients was correlated with a reduction in fractional anisotropy in this region. This study showed that a big dopaminergic loss must happen before related MRS measurements show a difference, proving the hypothesis that there is an onset of PD, and opening up the possibility of identifying potential PD sufferers before the onset of motor symptoms. Studies focusing on other ratios or metabolites should follow soon.

### Transcranial magnetic stimulation in parkinson’s disease

Transcranial magnetic stimulation is a novel tool in which a coil is placed over the intact skull (4). The coil induces electric fields in the cortex (a few centimeters in depth), which depolarize neurons in a region dependent on the shape of the coil and the current passing through it. If the pulses are triggered repeatedly and periodically, it is called repetitive TMS (rTMS). Recently, this approach has been used to treat different neurological and psychiatric disorders, such as epilepsy, attention deficit hyperactivity disorder, depression, and PD (4, 6, 55). The difficulty with this technique is the current lack of understanding of its after-effects, the variability between individuals, the different stimulation protocols, the range of available study designs, and so on (4, 6).

There is growing evidence that rTMS can be used to treat PD (4). Recently, TMS applications have focused on three general lines of research. First, as dyskinesia is thought to be associated with abnormal plasticity in the motor cortex, TMS techniques have been used to assess the effects of cortical plasticity (4). Second, the effects of TMS and rTMS on muscle function during gait and motion, as well as the effects of fatigue, have been investigated (4). Finally, the inhibitory effect of TMS techniques has been used to assess which brain regions are involved in the appearance of comorbidity, or behind a given symptom of PD (see Table 3). New information on cognition and different neurodegenerative disorders (e.g., PD, AD, or cognitive dysfunction) has been discussed elsewhere (56).

**Table 3:** **Magnetic resonance technology in Parkinson’s disease**.

Technique	Physical principle used	Use	Applications in PD
Anatomical magnetic resonance imaging (MRI)	Resonance signal of water content in tissues	Anatomical marker of pathologies and injures	Complements PD diagnosis excluding other
Diffusion tensor imaging (DTI)	Diffusion of water molecular in tissues	Marker of whit matter connections	Detecting white tract degeneration to use as biomarker
Resting state functional magnetic resonance (RS-MRI)	Low frequency oscillations of BOLD response	Indirect marker of neural connectivity	Study alterations of resting state networks due to PD. This aimed at finding a biomarker for diagnosis, as an indicator of treatment viability and as an indicator of the neurophysiology under symptoms from PD. Also used to study cortical plasticity
Functional magnetic resonance imaging (BLOD-fMRI)	Magnetic properties of blood	Indirect marker of neural activity (hemodynamic response)	Hypo–active Blood-fMRI signals in basal ganglia as well as motion brain structures for PD patients. Big influence of medications on result
Magnetic resonance spectroscopy (MRs)	Spectroscopic properties of chemical components of tissues	Marker of the amount of chemicals in tissues	Neuronal loss on basal ganglia assessed thought the NAA:Cr ratio. Increased GABA and decreased NAA:Cho, NAA:(Cho + Cr) for PD patients vs. Control
Magnetic resonance perfusion (MRP)	Flow and its characteristics in and outs tissues using RF pulses	Marker of: flow, transit time, time to peak and volume of blood to a tissue	Hypo–perfusion in same regions in which BLOD-fMRI presented hypo-activations for PD patients
Transcranial magnetic stimulation (TMS)	Induction of electrical and magnetic fields in a tissue using RF pulse	Activation and deactivation of brain regions	Assessment of the effects of cortical plasticity. Effect of TMS and rTMS on muscle function. Assessment of brain regions involved in PD symptoms production
Repetitive transcranial magnetic stimulation (rTMS)	As TMS, but repeated regulatory	As TMS but with regular repetitions	Assessment of the effects of cortical plasticity. Effect of TMS and rTMS on muscle function. Assessment of brain regions involved in PD symptoms production

*The first column shows MR techniques used for the diagnostic and research of PD. The second presents the physical properties of the technology used. The third, the use they have in general, and the fourth, specific applications in PD studies*.

For cortical plasticity, first, the effects of medication (usually levodopa) were assessed by comparing PD patients with and without dyskinesia. Cortical excitability after rTMS was similar for both groups, but inhibition was impaired in the dyskinesia group. This suggested that levodopa had an abnormal effect on cortical motor inhibitory circuits for these patients (57). Nevertheless, if treatment with rTMS was combined with treadmill training, it was shown that the effects on inhibitory circuitry were potentiated (58). It has been shown that low frequency rTMS delivered on consecutive days changed the excitability of motor cortex, by increasing the excitability of inhibitory circuits (59). The effects persisted for at least a day after rTMS. Applications of TMS for studying cortical plasticity in PD patients have produced conflicting results, as some studies showed increases in long-term potentiation at the neuronal level, while others have shown decreases the opposite result. Some authors have suggested that these differences arose from the chemical treatments patients were undergoing and also from the stage of the illness (60). Some work with rTMS included the development of new protocols like quadruple pulse stimulation (QPS), in which TMS pulses were separated by interstimulus intervals of 5 ms (QPS-5) and 50 ms (QPS-50). This setup induced bidirectional motor cortical plasticity. The short interval induced a potentiation effect, and the long interval a depression. Authors also reported a mediating effect of levodopa in their results (61).

The effects of TMS and rTMS on muscle function during gait and complex motion are also influenced by muscular fatigue. It is important to establish that the classical finger tapping test used for PD studies had to be limited to activations during comfortable performance of the task. If an extremely fast finger tapping movement was performed, then fatigue appeared as a constraint on the motor evoked potentials (MEPs) induced by TMS (62). The problem lay in the fact that this confound was not of cortical origin, so it was not a PD-related confound. A report, studying PD patients and comparing them to healthy subjects during TMS experiments (applied to the vertex), has shown that after contraction of the adductor muscle, normal subjects presented an initial gradual reduction in MEPs in their electromyography-silent period until the endurance point, and then an increase in these parameters. This effect was completely dissociated for PD patients who show impairment in the cortical compensatory mechanisms that healthy subjects have (63). Gait improvement (as well as cognitive function) was found in patients treated with rTMS in the dorsolateral prefrontal cortex (DLPFC), when combined with galantamine (64). The latter is an acetylcholinesterase inhibitor, which has a role in cognition as a memory modulator, and is known to reduce hallucinations in PD patients. The application of rTMS in the SMA has been found to improve handwriting in PD patients, ostensibly by changing the excitability of cortical networks throughout the brain (65). This last result was also supported by Maruo et al., who found that rTMS applied to the motor regions reduced the motor symptoms of PD patients (66). This was achieved over a single session, but several patients required the effect to be maintained for longer periods of time. Intermittent theta bursting (iTBS) has shown similar results to this work (67). In studies of rTMS on PD patients with dysarthria, treatment of the primary sensorimotor areas led to an improvement in voice quality and intensity, as well as an increase in speech rate and tongue movements (68).

In the third line of work addressed, the involvement of the cerebellothalamocortical (CTC) pathway that affects motion has also been assessed using TMS. This technique was applied to the primary motor cortex and the cerebellum of PD patients, and compared to results from a healthy group. Cerebellar inhibition was found to be reduced in PD patients. Rest tremors were affected by primary motor stimulation, while postural tremors were affected by both stimulations. This pointed toward involvement of different regions of the CTC in the transmission and generation of tremors (69). In other work by Nardone et al. (70), rTMS was applied to the DLPFC of PD patients with the compulsive disorder known as punding. The idea was to break the links between prefrontal structures and the striatum, which were thought to be responsible for these effects. The experiment was successful, and effects were found to be equivalent to those of levodopa in PD patients where the drug corrected this connection.

Progress using rTMS has been achieved through the use of animal models. In work by Hsieh et al., the scientists proved that a combination of rTMS and iTBS modulated motor activity in cortex, and suggested the possibility of using this model for treatment of early PD patients (71). They also found that this model worked only for animals, which had not yet lost the majority of their dopaminergic neurons in the substantia nigra, indicating that preservation of these neurons may be a biomarker for the usefulness of rTMS and TMS in general. Another murine model has been used to assess the therapeutic effects of rTMS on dopaminergic neurons of PD in animals (72). The treatment, when compared to controls which received sham rTMS, produced significant changes including improvement in treadmill locomotion and increased tyrosine hydroxylase-positive dopaminergic neurons, as well as dopaminergic fibers in striatum and substantia nigra. All this shows that rTMS has a neuroprotective function (73). New techniques like deep rTMS (rDTMS), which uses H-coils to allow for a deeper and more widespread stimulation, have been used to improve the Unified Parkinson’s Disease Rating Scale (UPDRS) scores significantly when stimulating primary motor and prefrontal cortex bilaterally (74).

Recently, there has been a trend toward combining TMS and rTMS with other techniques as BOLD-fMRI (75). In some interesting work, treatment with either fluoxetine or rTMS was given to depressed PD patients (76). The after-effects were assessed with BOLD-fMRI. This approach showed that, even when both treatments improved the mood disorder, different cortical areas were activated. rTMS increased activity in the left DLPFC and anterior cingulate gyrus, while fluoxetine did so in the right premotor and right medial prefrontal cortex. Both treatments affected the left medial prefrontal cortex but in different ways (effects were group and time dependent). This work shows that the different treatments had different effects on brain function and affected regions.

## Conclusion

Here, we have described novel MR and TMS techniques for studying PD. A summary of the results presented here can be seen in Table 3. The fact that these MR/TMS techniques are not as commonly used as DBS or normal structural MRI does not imply that they are ineffective; in fact, all of them contribute to the knowledge we have about PD. The BOLD-fMRI technique and perfusion MR have been focused on the regions known to be affected in PD (basal ganglia, motor, and prefrontal cortex). Both techniques showed hypoperfusion and smaller BOLD signals in these regions in PD patients, compared to controls. Spectroscopic findings worked mainly with the NAA-to-Cr ratio as an indicator of neuronal loss. Other chemical markers have not been used, but there are several ongoing investigations in this field. TMS has proven to be a useful tool to treat PD. It has been shown that it could be possible to knock-out a specific brain region for a given period of time, to assess which regions were correlated with comorbidity and separate different symptoms associated with the progression of PD. TMS techniques have also been used to assess cortical plasticity. Current research into future treatments for PD cannot be fully understood without including all available MR techniques.

Finally, we must remember that, however, exciting the neurobiological mechanisms might be the clinical usefulness of rational therapeutic approaches will be determined by their ability to provide efficacy, safety, and long-lasting and substantial improvements to quality of life. It is important that new studies are designed using criteria and end-point measures specific to different stages of PD, which will allow more definitive conclusions and understanding of this disease.

## Conflict of Interest Statement

The authors declare that the research was conducted in the absence of any commercial or financial relationships that could be construed as a potential conflict of interest.
